# Bacterial Effectors and Their Functions in the Ubiquitin-Proteasome System: Insight from the Modes of Substrate Recognition

**DOI:** 10.3390/cells3030848

**Published:** 2014-08-18

**Authors:** Minsoo Kim, Ryota Otsubo, Hanako Morikawa, Akira Nishide, Kenji Takagi, Chihiro Sasakawa, Tsunehiro Mizushima

**Affiliations:** 1 Division of Bacterial Infection Biology, Institute of Medical Science, The University of Tokyo, Shirokanedai 4-6-1, Minato-ku 4-6-1, Tokyo 108-8639, Japan; E-Mails: r-otsubo@ims.u-tokyo.ac.jp (R.O.); h-mrkw@ims.u-tokyo.ac.jp (H.M.); sasakawa@ims.u-tokyo.ac.jp (C.S.); 2 Picobiology Institute, Department of Life Science, Graduate School of Life Science, University of Hyogo, 3-2-1, Kouto, Kamigori-cho, Ako-gun, Hyogo 678-1297, Japan; E-Mails: rj12k019@stkt.u-hyogo.ac.jp (A.N.); ktakagi@sci.u-hyogo.ac.jp (K.T.); mizushi@sci.u-hyogo.ac.jp (T.M.)

**Keywords:** ubiquitin, effector, structure, pathogenic bacteria

## Abstract

Protein ubiquitination plays indispensable roles in the regulation of cell homeostasis and pathogenesis of neoplastic, infectious, and neurodegenerative diseases. Given the importance of this modification, it is to be expected that several pathogenic bacteria have developed the ability to utilize the host ubiquitin system for their own benefit. Modulation of the host ubiquitin system by bacterial effector proteins inhibits innate immune responses and hijacks central signaling pathways. Bacterial effectors mimic enzymes of the host ubiquitin system, but may or may not be structurally similar to the mammalian enzymes. Other effectors bind and modify components of the host ubiquitin system, and some are themselves subject to ubiquitination. This review will describe recent findings, based on structural analyses, regarding how pathogens use post-translational modifications of proteins to establish an infection.

## 1. Introduction

Infectious diseases caused by bacteria have significant impacts on human health. Many pathogenic bacteria, including *Shigella*, enteropathogenic *Escherichia coli* (EPEC), and enterohemorrhagic *E.coli* (EHEC), are associated with diarrheal diseases in young children and are an important cause of infant mortality [1,2]. Furthermore, the emergence of multidrug-resistant bacteria is an increasingly important threat around the world [3,4].

These pathogenic bacteria target human epithelial cells; various stimuli that arise during bacterial infection result in inflammatory responses, cell death, reorganization of the cytoskeleton, and exfoliation of epithelial cells [5]. To successfully colonize the intestinal epithelium, pathogenic bacteria possess highly specialized infection systems. Pathogenic bacteria such as *Shigella*, EPEC, and *Salmonella* deliver a variety of virulence factors, called effectors, into host cells via the type III secretion system (T3SS) [6]. In many cases, these effectors mimic or hijack host proteins and modulate host signaling pathways to promote bacterial infection [7,8].

Protein post-translational modifications (PTMs), such as phosphorylation, acetylation, ubiquitination, and deamidation, regulate many cellular processes and contribute to disease development [9]. PTMs modulate central signaling pathways in host cells, including mitogen-activated protein kinase (MAPK) cascades and the nuclear factor-kappa B (NFκB) and p53 regulatory pathways. Because PTMs are involved in many aspects of cellular physiology, many pathogenic bacteria target specific PTM pathways in various ways to inactivate host defense systems and establish infections [10]. Given the importance of overcoming pathogenic bacterial infections, we need to better understand the significance and underlying mechanisms of these PTM pathways. In this review, we discuss new discoveries about the PTM systems that are active during pathogenic bacterial infection. In particular, we focus on the structural recognition modes of bacterial effectors and their interactions with host substrates. These structural approaches provide us with atomic-scale molecular information about the effector-host interaction regarding the dynamics of the interaction, the modes of conformational changes due to protein modifications, and the determination of substrate specificities.

## 2. Exploitation of the Ubiquitin-Proteasome System by Pathogenic Bacteria

The ubiquitin-proteasome system (UPS) is an energy-dependent system that degrades specific target proteins. Ubiquitin is a highly conserved small protein that is covalently attached to substrate proteins. The covalent attachment of ubiquitin (called ubiquitination) requires a cascade of three enzymatic reactions. First, a ubiquitin-activating enzyme (E1) forms a thioester bond with ubiquitin in a reaction that requires ATP. Next, E1 delivers ubiquitin to a ubiquitin-conjugating enzyme (E2). Finally, ubiquitin ligase (E3) transfers ubiquitin from E2-ubiquitin to the lysine residue of a substrate protein [11]. Generally, ubiquitin becomes attached to one or more lysine residues of a target protein and forms mono- or poly-ubiquitin chains. Threonine, serine, cysteine, and N-terminal methionine can also be modified by ubiquitin. Ubiquitin contains seven lysines and can form eight types of poly-ubiquitin chains (K6, K11, K27, K29, K33, K48, K63, and M1), which are used as molecular signals for various cellular pathways [12,13]. For example, K48-linked chains mark proteins for degradation by the proteasome, whereas K63-linked chains mediate signal transduction. Ubiquitination is a reversible reaction; deubiquitinating enzymes (DUBs) remove ubiquitin from substrates and recycle the ubiquitin [14]. Ubiquitination plays a fundamental role in many cellular processes, including cell cycle progression, transcriptional control, protein trafficking, signal transduction, immune responses, cancer, and infectious diseases [15,16,17,18,19,20].

Recent studies have identified several bacterial effectors that interact and modulate the UPS during infection by pathogenic bacteria (summarized in Table 1) [21].

**Table 1:** Summary of protein post-translational modifications (PTM)-targeting bacterial effectors.

Function		Pathogens	Effector	Targets	Mechanism of Action	Ref
ubiquitin ligase (E3)	HECT-like	enterohemorrhagic *E.coli* (EHEC)	NleL	Unknown	Pedestal formation	[22,23]
		*S.* Typhimurium	SopA	Unknown	Regulation of inflammation	[24,25]
	RING/U-box	EHEC	NleG	Unknown	Unknown	[26]
		*L. pneumophila*	LubX	Clk1, SidH	Unknown	[27,28]
		*P. syringae*	AvrPtoB	Fen, CERK1, FLS2, BAK1	Suppression of plant immunity	[29,30,31,32]
	Novel E3 ligase (NEL)	*Rhizobium*	NopM	Unknown	Unknown	[33]
		*S.* Typhimurium	Slrp	Trx, ERdj3	Induction of cell death Interference with unfolded protein response	[34,35]
			SspH1	PKN1	Inhibition of androgen receptor signal	[36,37]
			SspH2	NOD1, SGT1	Up-regulation of inflammation	[38]
		*Shigella*	IpaH1.4	Unknown	Unknown	[39]
			IpaH3	Unknown	Unknown	[40]
			IpaH4.5	p65	Suppression of inflammation	[41]
			IpaH9.8	Ste7, U2AF ^35^, NEMO	Suppression of inflammation/splicing	[42,43,44]
			IpaH0772	TRAF2	Suppression of inflammation	[45]
Kinase		*Shigella*	OspG	UbcH5	Suppression of inflammation	[46,47]
		EHEC	OspG	Unknown	Unknown	[48]
		*Y. enterocolitica*	OspG	Unknown	Unknown	[48]
Deamidase		*Shigella*	OspI	Ubc13	Suppression of inflammation	[49,50,51]
		enteropathogenic *Escherichia coli (*EPEC)/EHEC	Cif	NEDD8, Ub	Inhibition of Cullin-RING ligases (CRL) activation	[52,53,54,55,56]
		*B. pseudomallei*	CHBP	NEDD8, Ub	Inhibition of CRL activation	[52,57,58,59]
		*P. luminescens*	Cif_Pl_	NEDD8	Inhibition of CRL activation	[57]
		*Y. pseudotuberculosis*	Cif_Yp_	NEDD8, Ub	Inhibition of CRL activation	[60]

**Figure 1:**
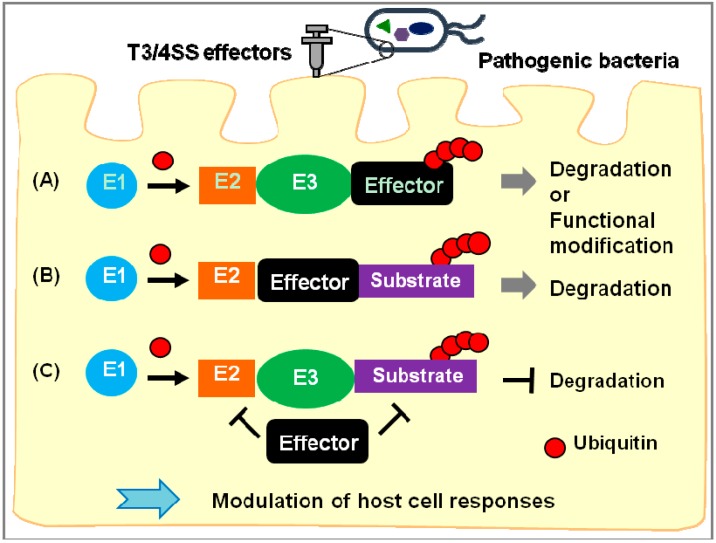
Bacterial effectors manipulate the host ubiquitin-proteasome system (UPS). After translocation into host cells, several effectors (black squares) target the host’s UPS. (**A**) Some effectors are ubiquitinated and then either degraded by the proteasome or functionally modified; (**B**) Some effectors, such as ubiquitin ligases (E3s), act as UPS enzymes; (**C**) Other effectors inhibit specific UPS steps. Bacterial effectors can mimic or hijack the host’s UPS to modulate host cell responses such as immune responses, cell death, and clearance of bacteria.

These effectors are classified into three types (Figure 1): (A) effectors that are targeted by the host ubiquitin ligases and are quickly inactivated by degradation or modified by ubiquitination, thereby diversifying effector function; (B) effectors with UPS enzyme activity, such as E3 ubiquitin ligase and DUB activity; and (C) effectors that modulate steps of the UPS. Examples of each type follow: (A) The *Salmonella* effectors SopE and SptP are degraded sequentially by ubiquitination to regulate bacterial invasion [61]. *Yersinia* YopE is ubiquitinated, and degradation of YopE contributes to the spread of bacteria in host tissues [62,63]. However, the host or bacterial E3 proteins that ubiquitinate these effectors are still unknown. Ubiquitination of *Salmonella* SopB diversifies its function by altering its subcellular localization [64,65]. (B) *Salmonella* SseL exhibits DUB activity *in vitro* and is required for *Salmonella* infection in mice [66]. *Salmonella* SopA and EHEC NleL are structurally similar to mammalian HECT E3 ligases, although their substrates are unknown [22,23,24,25]. Crystallographic analysis has revealed that the *Pseudomonas syringae* effector AvrPtoB, which suppresses immune responses triggered by pathogen-associated molecular patterns, is a structural mimic of the RING family of E3 ligases [67]. AvrPtoB binds to several host kinases such as Fen kinase and BRI1-associated kinase1 (BAK1), which are important for plant immunity [68], resulting in their ubiquitination and degradation [29,30,31,32]. (C) The EPEC effector NleE methylates TAB2 and TAB3, an activator of the NFκB pathway [69], and this modification blocks host NFκB signaling [70]*.* The *Shigella* effector IpaB binds to Mad2L2, an APC inhibitor, to inhibit host cell-cycle progression [71].

In addition to the various types of effectors, we will also discuss recent structure-based discoveries that reveal the modes of molecular recognition between bacterial UPS target effectors and their substrates, as well as how these modifications influence pathogenesis.

### 2.1. Novel E3 Ligases and Their Substrates

The IpaH family is highly conserved among many pathogenic bacteria, including *Yersinia*, *Salmonella*, *Edwardsiella*, *Bradyrhizobium*, *Rhizobium*, and some *Pseudomonas* species [44]. The members of this family share a high degree of structural and functional similarity with *Shigella* IpaH proteins. Members of the IpaH family contain a leucine-rich repeat (LRR) involved in substrate recognition and an E3 ligase domain in the C-terminal region (CTD). Crystal structures have revealed that the ubiquitin-ligase domain structure of these IpaH proteins is distinct from well-known E3 structures such as the HECT and RING finger domains, and has therefore been termed “Novel E3 ligase” (NEL) [39,40,72]. Several recent studies have reported identification of host proteins ubiquitinated by NEL family proteins. For example, *Shigella* IpaH9.8 ubiquitinates the NFκB modulator (NEMO)/IKKγ, an essential component of the IκB kinase complex. As a result, NEMO is degraded by the host proteasome, and NFκB activation and subsequent inflammatory response to *Shigella* infection are attenuated [42]. Another *Shigella* effector, IpaH0722, targets TNF receptor-associated factor 2 (TRAF2), an adaptor protein involved in NFκB signaling, and thereby decreases protein kinase C-mediated NFκB activation during *Shigella* infection [45].

*Salmonella* delivers SspH2, a NEL family member, via T3SS Pathogenicity Islands 2 (SPI-2), which is localized to the plasma membrane by S-palmitoylation of a conserved cysteine residue in its N-terminal domain [73]. SspH2 interacts with nucleotide-binding oligomerization domain-containing protein 1 (NOD1) and its co-chaperone, suppressor of G2 allele of Skp1 (SGT1). SspH2 mono-ubiquitinates NOD1 and increases NOD1 activity, thereby modulating innate immunity [38]. However, it is unclear how SspH2 structurally and biochemically recognizes NOD1 during *Salmonella* infection.

**Figure 2:**
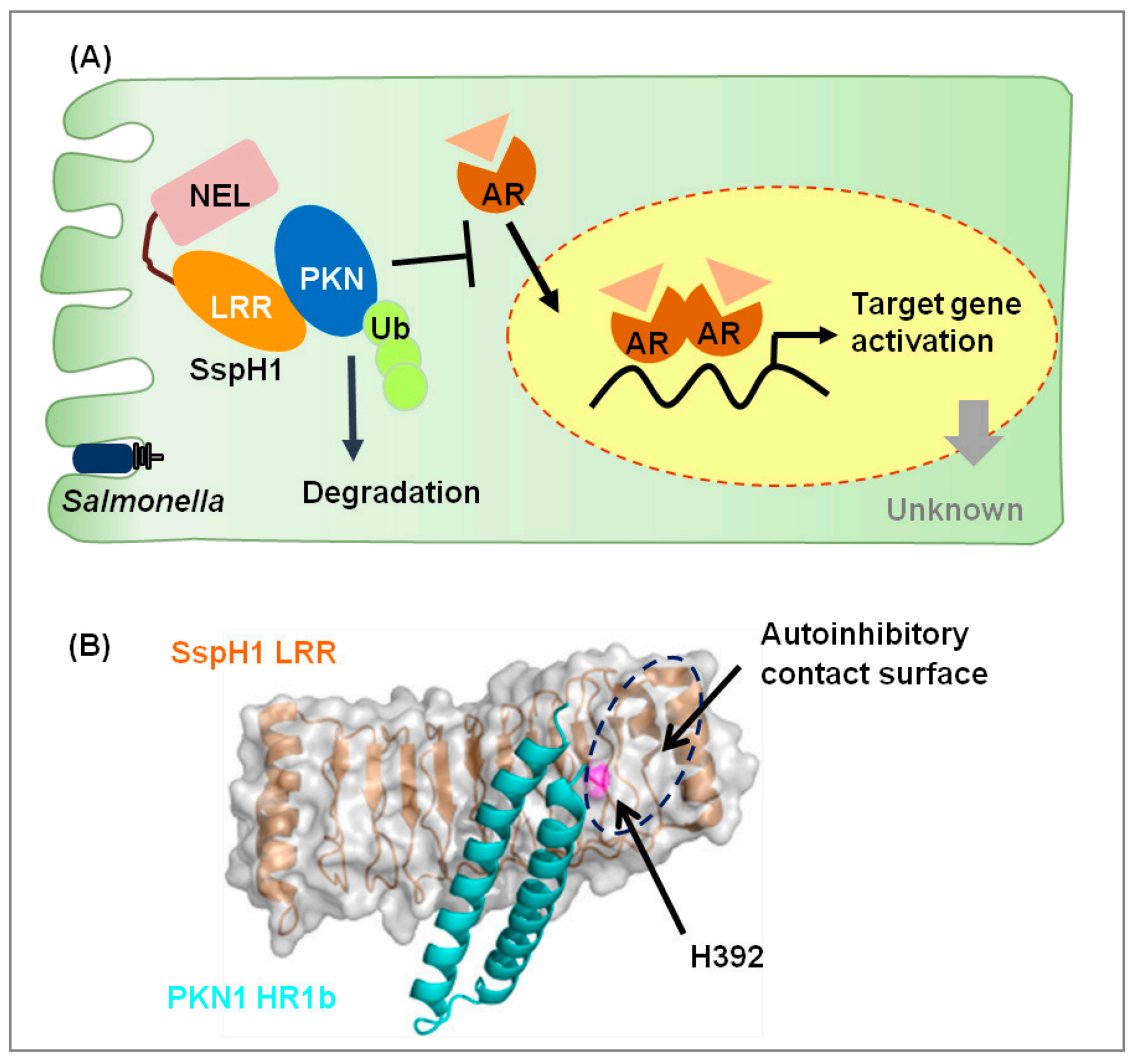
Novel E3 ligase (NEL) family ubiquitin ligases. (**A**) SspH1 is secreted into host cells by *Salmonella* T3SS SPI-1 and SPI-2. The leucine-rich repeat (LRR) domain of SspH1 recognizes and interacts with the homology region 1b (HR1b) coiled-coil subdomain of protein kinase N1 (PKN1). PKN1 is ubiquitinated and degraded by SspH1, resulting in down-regulation of androgen receptor (AR) signaling. In the cytoplasm, AR (in complex with heat shock proteins 70 and 90) interacts with ligands such as testosterone, and then translocates to the nucleus to act as a transcription factor; (**B**) SspH1-PKN1 interaction surface.The figure illustrates the crystal structure of the LRR of SspH1 in complex with the HR1b of PKN1 (cyan) (PDB ID 4NKG). Protein surface and cartoon representation of SspH1 are shown in gray and orange, respectively. H392 of SspH1 is shown in magenta. The contact interface between LRR and NEL is indicated by a dashed oval. The molecular graphics were prepared using PyMOL [76].

*Salmonella* SspH1, another NEL family member, induces protein kinase N1 (PKN1) degradation in a ubiquitin- and proteasome-dependent manner [36]. PKN1 interacts with androgen receptor (AR), functions as a transcriptional coactivator, and regulates a wide range of cellular processes including cytoskeletal regulation, tumorigenesis, and transcription [74,75]. In 293T cells, expression of wild-type SspH1, but not E3 ligase-dead or SspH1-PKN1 interface mutants, suppresses the AR-responsive element [37]. Degradation of PKN1 by SspH1 attenuates the AR response (Figure 2A).

The interaction between SspH1 and human PKN1 is one of the best-characterized models of substrate recognition by a NEL ligase (Figure 2B). The homology region 1b (HR1b) subdomain of PKN1 directly interacts with the LRR domain of SspH1 [37]. The structure of the SspH1^LRR^-PKN1^HR1b^ complex has revealed the interaction mode and the mechanism of substrate-mediated activation of the ligase.

The structure of each protein in the SspH1^LRR^-PKN1^HR1b^ complex is similar to that of the corresponding free protein. The LRR domain of SspH1 interacts specifically with the HR1b coiled-coil sub-domain of PKN1; this interaction is mediated by a mixture of hydrogen bonds, salt bridges, and hydrophobic interactions involving residues that are not conserved across the family [37]. A C-terminal helix α2 of PKN1 is critical for the interaction with the LRR domain of SspH1 [37]. Structural studies of IpaH3 and SspH2 suggested that these effectors have auto-inhibitory states that are mediated by LRR-NEL interactions [77,78]. Based on the structure of this complex, the substrate and NEL domains appear to bind to the same interface of the LRR domain, indicating that the two interactions are mutually exclusive. In this class of bacterial ubiquitin ligases, the mechanisms of substrate recognition and auto-regulation are coupled. However, many crucial aspects concerning the mode of action, physiological roles, and targets of this important ubiquitin ligase family remain to be elucidated.

### 2.2 The Deamidase OspI Targets Ubc13

**Figure 3:**
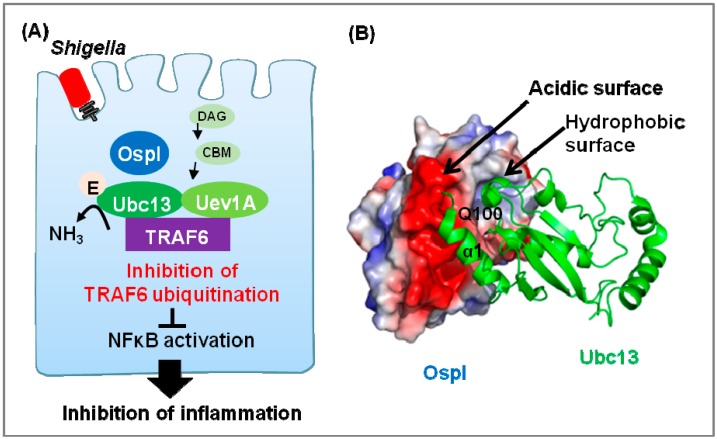
Bacterial deamidase OspI targets Ubc13. (**A**) The *Shigella* effector OspI deamidates Ubc13 to inhibit TRAF6 activation, thereby inhibiting host immune responses; (**B**) Surface-potential representation of OspI and Ubc13 (PDB ID 3W31). Red, blue, and white represent acidic, basic, and neutral, respectively. The complementary surface patches of OspI responsible for complex formation are labeled. Q100 of Ubc13 is shown as a stick model.

Invasive pathogens such as *Shigella* and *Listeria* induce membrane disruption [79]. This irregular membrane rupture, which is sensed as damage-associated molecular patterns (DAMPs), rapidly activates many cellular signals and triggers the host inflammatory responses [80]. *Shigella* invasion is sensed as DAMPs, which activate a signaling pathway involving diacylglycerol, the CBM (CARD-Bcl10-Malt1) complex, TNF receptor-associated factor 6 (TRAF6), and NFκB [51]. *Shigella* secrets OspI into host cells via the T3SS at this stage of infection. OspI specifically binds to Ubc13, an E2 enzyme, and modifies this enzyme by deamidating Gln100 to Glu100. This modification inhibits Ubc13’s E2 ubiquitin-conjugating activity, which is required for TRAF6 ubiquitination and activation of the diacylglycerol-CBM-TRAF6-NFκB signaling pathway (Figure 3A). As a result, in HeLa cells infected with wild-type *Shigella*, but not in cells infected with an *ospI* deletion strain, expression of various chemokines and cytokines is suppressed. Consistent with these data, guinea pig rectum infected with an *ospI* deletion strain exhibited elevated inflammatory responses [81]. The crystal structures of OspI alone and OspI C62A in complex with Ubc13 have been determined (Figure 3B) [49,50,51]. OspI has an α/β fold with four β-strands, seven α-helices, and one 3_10_ helix; it also contains a putative Cys-His-Asp catalytic triad that is conserved in the cysteine protease superfamily. Mutational analysis of these residues has demonstrated that the catalytic triad is essential for OspI’s enzymatic activity.

In the structure of the complex, the interface between OspI and Ubc13 consists of a hydrophobic surface and a complementary charged surface. The charged interaction involves an acidic patch of OspI and the positively charged α1 helix of Ubc13. Relative to the structure of Ubc13 alone, the α1 helix of Ubc13 re-orients upon OspI binding. Additional mutational analysis has confirmed that the α1 helix is critical for OspI’s ability to discriminate between host E2 enzymes. In the crystal structure of wild-type OspI alone, the Sγ position of the active-site Cys62 is located on the opposite side of the active site. In the complex, however, this residue has undergone significant conformational changes. This conformational change in the active site Cys62 is also observed in the uncomplexed OspI C62A mutant. The catalytic His and main-chain atoms of the Cys (Ala for OspI C62A) and Asp residues align well between OspI C62A and canonical cysteine proteases. In the complex, Gln100 of Ubc13 protrudes into the active-site cleft.

Ubc13 forms a heterodimer with its E2 variant, Uev1a, and this heterodimer interacts with specific ubiquitin ligases of TRAF6 and induces K63-linked polyubiquitination [82]. Uev1a is located at one end of Ubc13, whereas OspI is located at the other end. TRAF6 interacts with Ubc13 through the α1 helix and two loops, including Gln100, which are the same regions involved in OspI binding. The deamidation of Gln100 may decrease the affinity of TRAF6 for Ubc13.

### 2.3. The Deamidase Cif Targets Ubiquitin or NEDD8

**Figure 4:**
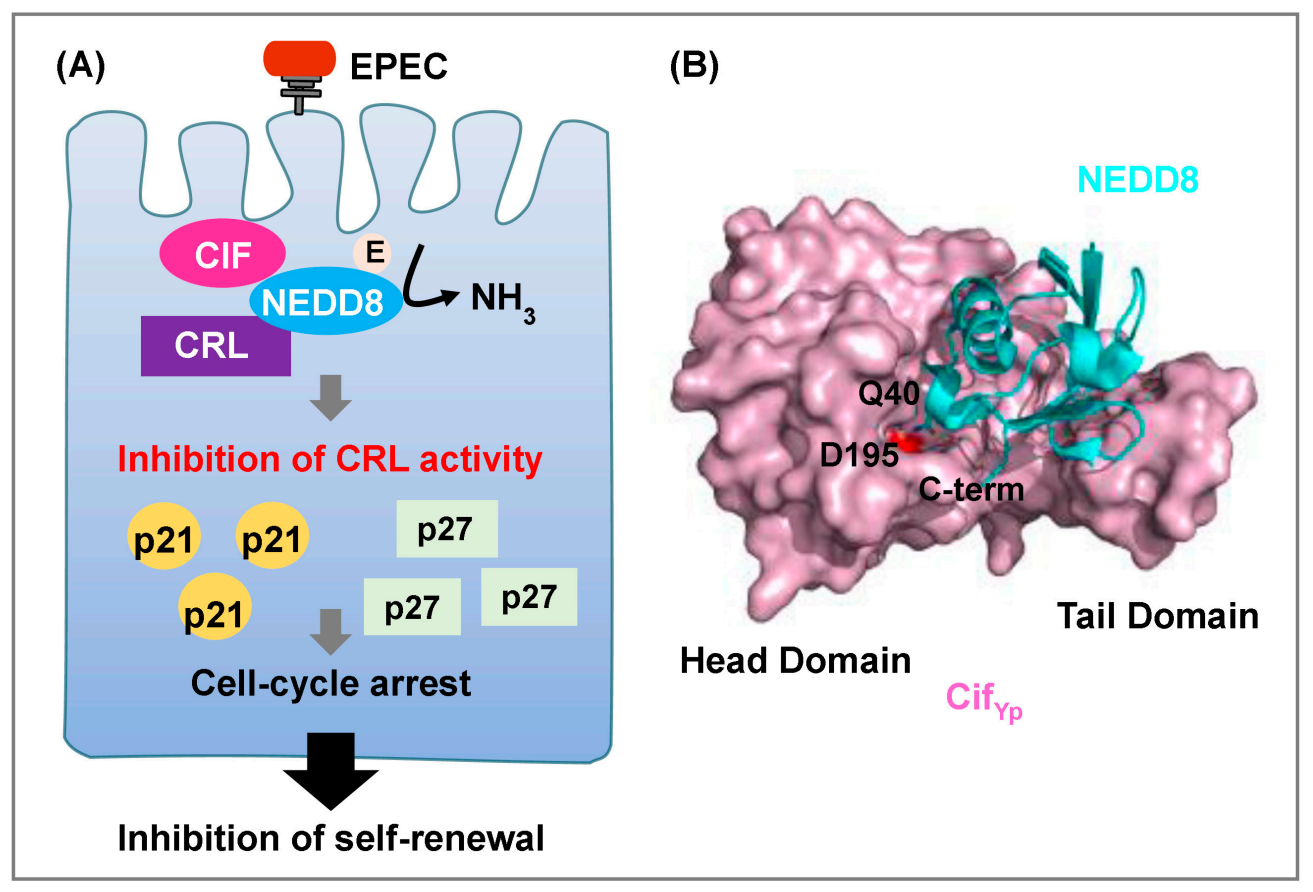
Bacterial deamidase Cif family proteins target host UPS. (**A**) The enteropathogenic *Escherichia coli* (EPEC) effector Cif deamidates NEDD8 to inhibit CRL activity. Deamidation of NEDD8 results in G1 or G2/M cell-cycle arrest via accumulation of the cyclin-dependent kinase inhibitors p21 and p27 in epithelial cells; (**B**) Surface representation of Cif_Yp_ (pink) complex with NEDD8 (cyan) (PDB ID 4F8C). D195 of Cif_Yp_ is shown in red. Q40 of NEDD8 is shown as a stick model.

The cycle-inhibiting factors (Cifs) are a family of papain-like type III effector proteins conserved in Gram-negative pathogenic bacteria such as EPEC, EHEC, *Yersinia pseudotuberculosis*, *Photorhabdus luminescens*, and *Burkholderia pseudomallei* [83]. Crystallographic analyses have shown that Cif family proteins, like OspI, also contain a Cys-His-Gln catalytic triad [53,57,59]. Cif family proteins induce host cell-cycle arrest by deamidating a specific glutamine residue in their substrates. Cif from EPEC (Cif_Ec_) and Cif from *Burkholderia pseudomallei* (CHBP) recognize their host-encoded targets, ubiquitin (Ub) and neural precursor cell-expressed developmentally downregulated 8 (NEDD8), and specifically deamidate Gln40 in their target proteins to Glu40. This modification of NEDD8 impairs host protein degradation by inhibiting Cullin-RING ligases (CRLs), leading to accumulation of CRL substrates during EPEC infection (Figure 4A) [52]. Consequently, EPEC-infected cells undergo cell-cycle arrest, late apoptosis (in epithelial cells), and apoptosis (in macrophages) [54,55,56]. Furthermore, ectopic expression of NEDD8 (Q40E) in epithelial cells causes the same phenotypes as EPEC infection [52].

Structures of Cif proteins reveal that the family members have similar 3D-folds consisting of a head-and-tail domain arrangement. Structures of Cif from *Yersinia pseudotuberculosis* (Cif_Yp_), Cif from *Photorhabdus luminescens* (Cif_Pl_), and CHBP in complex with NEDD8 have shown how Cif family proteins engage their substrates and catalyze the deamidation of NEDD8 (Figure 4B) [58,60]. These complexes have similar overall structures. The structure of the Cif-NEDD8 complex shows that both the C-terminal head domain and the N-terminal tail domain of Cif make significant contributions to the NEDD8-binding interface. NEDD8 forms an interface with Cif at four regions, and these interactions induce a slight reorientation at the tip of the tail domain of Cif that bends toward NEDD8. In NEDD8, the C-terminal residues shift upon binding to Cif. In the complex, Gln40 of NEDD8 positions the catalytic center of Cif. Based on the structure of the complex, it is likely that specific hydrogen bonds between Cif and NEDD8 contribute to the selection of the deamidation site. For example, Asp195 (Cif_Yp_ numbering), a residue conserved in all Cif family proteins, forms a hydrogen bond with Nε2 of Gln40 in NEDD8.

Cif family proteins catalyze NEDD8 deamidation more efficiently than Ub deamidation. The crystal structures of CHBP in complex with NEDD8 and Ub, and unbiased molecular dynamics (MD) simulations of these complexes, have suggested that Ub is more mobile than NEDD8 in the CHBP complex [58]. ITC and mutational analyses have shown that Glu31 in NEDD8 mediates an electrostatic interaction that is a critical determinant of CHBP’s substrate preference for NEDD8 [58]. The important amino acids involved in substrate recognition are well conserved between Ub and NEDD8, but not in other ubiquitin-like proteins. These interactions may determine the specificity of substrate recognition.

CRL complexes consist of seven different Cullin proteins (Cullin1, 2, 3, 4A, 4B, 5, and 7) [84]. All these Cullins are neddylated, and neddylation is important for CRL activity. Future studies should investigate how these different CRLs are regulated by Cif family proteins during pathogenic bacterial infections. Targeting eukaryotic proteins for deamidation is a general mechanism of bacterial virulence.

### 2.4. Kinase OspG Regulates E2

**Figure 5:**
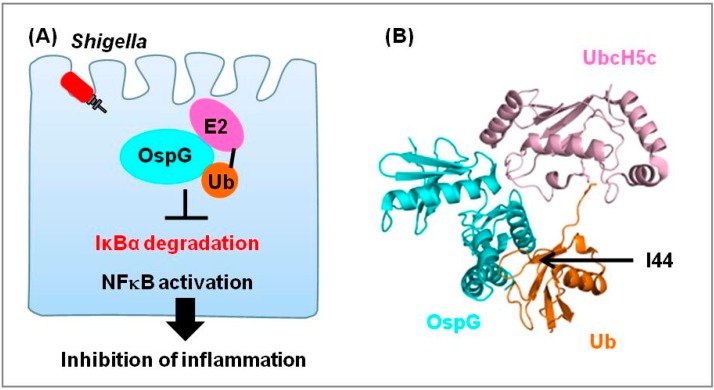
The *Shigella* effector OspG targets E2 enzyme. (**A**) OspG is activated by binding to host E2~Ub. This complex inhibits the host inflammatory response during *Shigella* infection; (**B**) Crystal structure of the OspG-UbcH5c-O~Ub complex. OspG, UbcH5c, and Ub are shown in cyan, pink, and orange, respectively (PDB ID 4BVU). I44, at the center of the hydrophobic surface, is indicated with an arrow.

Several bacterial kinases manipulate or disrupt host signaling pathways involving protein phosphorylation [85]. *Shigella* delivers the OspG effector, which functions as a serine/threonine kinase, and appears to exploit both eukaryotic ubiquitin and phosphorylation signaling pathways [46]. *Shigella* OspG contains conserved catalytic motifs found in mammalian kinases, and it also undergoes autophosphorylation. OspG binds to several E2 proteins and E2-ubiquitin. During *Shigella* infection, OspG inhibits IκBα degradation and NFκB activation; consistent with this, rabbit ileal loops infected with an *ospG* deletion mutant exhibited strong inflammatory immune responses (Figure 5A). OspG exerts kinase activity and interacts directly with E2-Ub conjugates.

Recently, the crystal structure of the OspG-UbcH5c-O~Ub complex (covalent oxyester linkage between the UbcH5c active site and the Ub C-terminus) was determined at a resolution of 2.7 Å (Figure 5B) [47]. In the complex structure, binding to OspG does not induce significant conformational changes in either UbcH5c or Ub. Both UbcH5c and Ub contact OspG, but bind to distinct surfaces, placing the Ub and UbcH5c in an extended conformation with respect to each other. Ub binding to OspG involves a hydrophobic surface centering on I44. UbcH5c sterically distant with respect to interactions occurs primarily in the N-terminal lobe of OspG, whereas Ub binds to the C-terminal lobe.

OspG contains a minimal kinase domain. Whereas canonical kinases have C-terminal α-helical elements, in the OspG-UbcH5c~Ub complex, the C-terminal region of OspG is occupied by the Ub subunit. UbcH5c~Ub binding stabilizes an active conformation of the kinase, and complex formation is essential for maximal OspG activity. Moreover, the E2~Ub in the OspG-E2~Ub complex is less reactive than E2~Ub in solution. Formation of the OspG-UbcH5c~Ub complex promotes kinase activity while reducing reactivity of the E2~Ub conjugate. This structural analysis provides atomic-level details regarding the mode of OspG kinase activation, and illustrates the kinase-regulation mode of E2~Ub conjugates, a novel function of E2~Ub. In oral infections of mice, an *ospG* strain with a mutation in the OspG-E2~Ub interface exhibits a phenotype similar to that of an *ospG* deletion strain. In addition, a kinase-dead mutant of OspG also exhibits elevated inflammatory responses *in vivo*. These data indicate that kinase activity and the binding of E2~Ub are important for establishment of shigellosis. *ospG* homologues are present not only in *Shigella* but also EHEC and *Yersinia enterocolitica*; these proteins have highly conserved structures [48]. However, colons infected by an EHEC *ospG* deletion strain do not exhibit a severe inflammatory response. Future studies should identify the target proteins of each OspG homologue in order to provide insight into these pathophysiological differences.

## 3. Conclusions

The PTMs that occur during bacterial infection represent key biological processes, both in regard to understanding the molecular basis of bacterial infection and in the context of controlling infectious disease. In this review, we described several bacterial effectors that affect specific steps in the ubiquitination pathway. *Shigella* effectors such as OspI and OspG interact with host E2 enzymes and interfere with their function. Several bacterial effectors mimic E3 ligases, such as NEL family proteins, to promote ubiquitination-mediated degradation of their own specific targets, thereby promoting infection. In addition, Cif family proteins target ubiquitin or ubiquitin-like proteins for deamidation in order to inactivate their function; deamidation is a pathogenic strategy used by many bacterial pathogens.

As mentioned above, the emergence of multidrug-resistant bacteria is a serious problem worldwide. To overcome the threat of pathogenic bacterial infection, we need to better understand the molecular mechanisms of bacterial infection and develop new antibiotics or vaccines based on novel strategies and drug targets. After the successful development of the proteasome inhibitor bortezomib, general components of the ubiquitin system have been recognized as potential drug targets [86]. The characterization of pathogen-encoded enzymes that catalyze specific PTMs critical for infection will provide new targets for development of effective drugs. Structural analyses of interaction modes have enabled *in silico* screening of small molecules that inhibit effector-substrate interactions, and such analyses should also be useful for the precise design of selective and effective drugs. Unfortunately, the host targets of most of these bacterial effectors are currently unknown. Therefore, future studies should focus on identifying the actual target(s) of these effectors and on comprehensively determining the structure of effector-substrate complexes.

Furthermore, these effector proteins, e.g., IpaH and Cif, are highly conserved among many pathogenic bacteria, including EHEC, *Salmonella*, and *Yersinia*, and family members share extensive structural and functional similarities. The discovery of small molecules that inhibit their enzymatic activities should also provide effective therapeutic tools against several diseases caused by infectious bacteria.
